# Increased gaze cueing of attention during COVID-19 lockdown

**DOI:** 10.1016/j.isci.2021.103283

**Published:** 2021-10-15

**Authors:** Mario Dalmaso, Luigi Castelli, Giovanni Galfano

**Affiliations:** 1 Department of Developmental and Social Psychology, University of Padova, 35131 Padova, Italy

**Keywords:** Cognitive neuroscience, Psychology, Social interaction, Social sciences

## Abstract

Affiliation with others is a basic human need. The lockdown implemented for counteracting the COVID-19 pandemic has determined an unprecedented situation of social deprivation, forcing individuals to dramatically reduce face-to-face interactions. This, in turn, has caused relevant consequences on psychological well-being. However, the impact of lockdown-related social isolation on basic cognitive processes is still largely unknown. Here, we focus on social attention and address gaze cueing, namely the ability to shift attention in response to the gaze of others. This is a hard-wired cognitive mechanism critically supporting the establishment of social interactions and pervasive relationships among individuals. Our results show a stronger gaze-cueing effect during, rather than after, the lockdown, whose magnitude was positively correlated with social isolation distress. These findings indicate that, in a condition of prolonged social deprivation, orienting of attention may be shaped by hypersensitivity to social cues, likely due to the strive to reconnect with others.

## Highlights

• The impact of COVID-19 lockdown on cognitive processes is still largely unexplored
• We focus on gaze cueing of attention as a building block of social interaction
• Gaze cueing was larger during the lockdown than after the lockdown
• Social deprivation is associated with increased sensitivity to the eyes of others

## Graphical abstract

**Figure:**
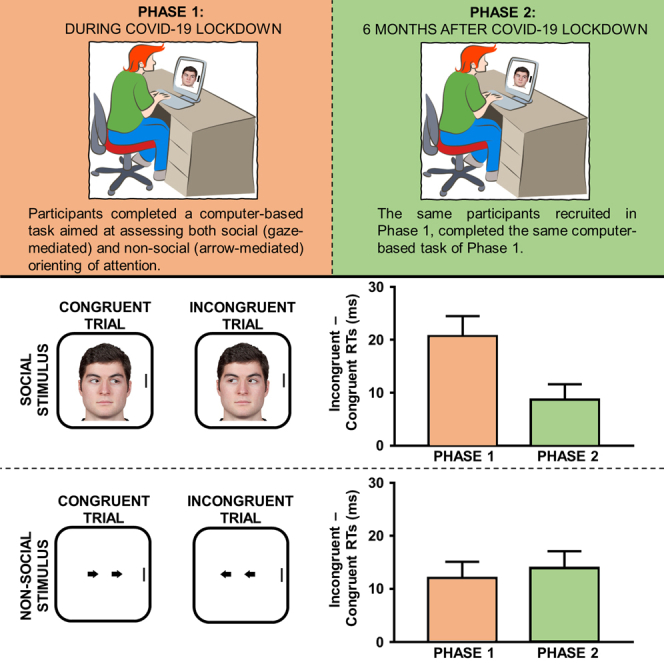

## Introduction

Italy was the first country to suffer a major COVID-19 emergency in Europe (Lavezzo et al., 2020). In the first months of 2020, COVID-19 spread all over the world and, on March 11th, the World Health Organization (WHO) declared COVID-19 a global pandemic (WHO, 2020). Even before that date, on March 9^th^, the Italian government imposed—first case among Western nations—a national lockdown to curtail the spread of the virus. For 56 days (until May 4^th^), citizens were strictly forbidden to leave their houses (except for primary needs or health reasons), thus leading a whole nation to a widespread and long-lasting condition of social isolation at home.

Under a psychological perspective, humans display an intrinsic need to establish social interactions, and our daily life is generally characterized by continuous social exchanges with others (Baumeister and Leary, 1995; Tomasello, 2014). In this regard, although the lockdown was effective in preventing the spread of the virus (Silverio et al., 2020), it also led to undesirable side effects on psychological well-being (Sibley et al., 2020).

Much less is known concerning lockdown-driven effects on more basic cognitive mechanisms supporting social interaction. The lockdown provided a unique opportunity to evaluate the effects of a sudden, unexpected, and dramatic reduction of face-to-face social exchanges on the basic cognitive mechanisms subtending our social behavior. Among these, social attention, namely the hard-wired ability to orient one's attention in response to social signals provided by others, represents one of the most relevant building blocks shaping social cognition, involved in perspective taking and creating shared representations of the environment (Capozzi and Ristic, 2018; Dalmaso et al., 2020a; Emery, 2000). More specifically, it has been widely documented that people tend to orient their attentional resources toward the same spatial location gazed at by another individual. Experimentally, this is often investigated by employing a paradigm called gaze-cueing task (Driver et al., 1999; Friesen and Kingstone, 1998), in which participants respond to a lateralized target while ignoring a task-irrelevant central face with averted gaze. Typically, an increased performance (i.e., smaller response latencies and a greater accuracy) is reported when the target appears on the same spatial location gazed at by the face (i.e., spatially congruent trials) relatively to other spatial locations (i.e., spatially incongruent trials). This is taken as evidence that averted-gaze stimuli can induce attentional shifts in an observer, namely the gaze-cueing effect (Driver et al., 1999; Friesen and Kingstone, 1998).

The impact of a genuine condition of social isolation on social attention, such as that experienced during the lockdown, is unknown, and its study could provide novel and important insights for understanding how a prolonged deprivation of social interactions can impact on crucial attentional mechanisms. To this aim, we developed a gaze-cueing task and delivered it to the same sample of individuals both during and after the lockdown. Arrows, namely other stimuli known to elicit reliable attention shifts (Dalmaso et al., 2020b; Tipples, 2002), were also employed as nonsocial, control cueing stimuli. In so doing, we aimed to identify the selective effects of social isolation on gaze cueing. Previous research has shown that experiences of social isolation can have significant effects on the subsequent search for social interaction in both humans (Gardner et al., 2005) as well as other animal species (Latané and Steele, 1975). Moreover, it has recently been reported that social isolation can lead to a brain activation response to social interaction scenes similar to that observed in starving individuals when exposed to food images (Tomova et al., 2020). This craving-like response for socially deprived individuals has been interpreted as evidence that social affiliation is a built-in need.

**Figure 1:**
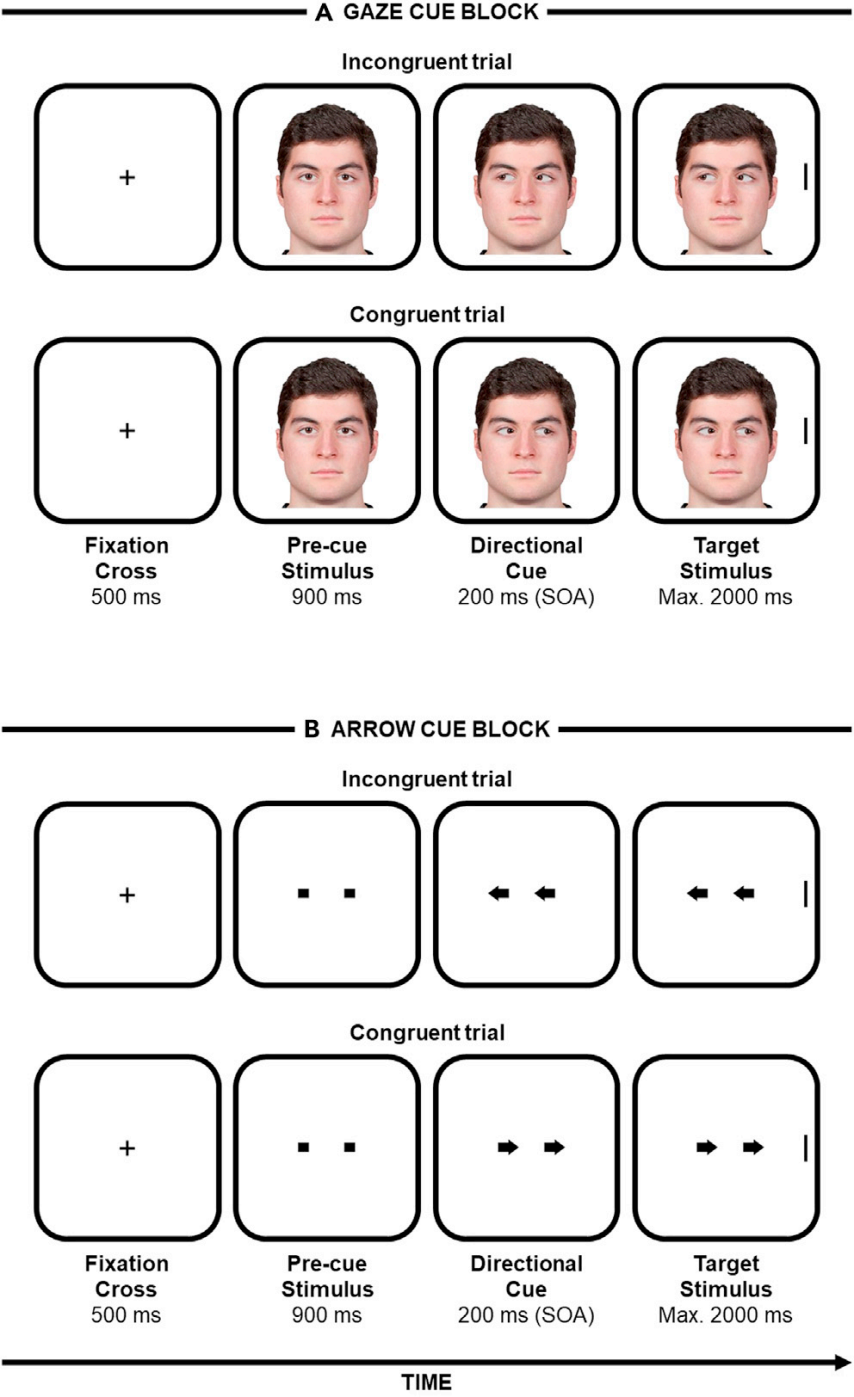
The spatial-cueing task Examples of the stimuli and trials. Participants were instructed to look at the center of the screen for the whole duration of the trial, to ignore gaze/arrow direction because it was not informative as concerns the location of the upcoming target, and to discriminate—as fast and accurately as possible—the orientation (vertical versus horizontal) of the target by means of a manual keypress. Please note that in the control experiment only the gaze cue block was administered. (A) Examples of an incongruent and a congruent trial with gaze stimuli. (B) Examples of an incongruent and a congruent trial with arrow stimuli.

Moving from these conceptual premises, participants in the present study took part in two distinct phases. They completed the first phase (N = 104) of the experiment after about eight weeks of lockdown (from April 28^th^ to May 3^rd^ 2020) and before its end. In so doing, we were able to test individuals who had been living in a condition of social isolation for almost two months. Then, they were invited to complete the second phase of the experiment after a period of about six months, in which no lockdown restrictions were active (from October 19^th^ to October 28^th^ 2020). The final sample, which included participants who completed the task in both phases, was composed of 67 individuals. In both phases, the participants were requested to take part in a spatial-cueing task with gaze and arrow stimuli serving as noninformative social and symbolic control cues, respectively (see Figure 1). The whole experiment was delivered online.

**Figure 2:**
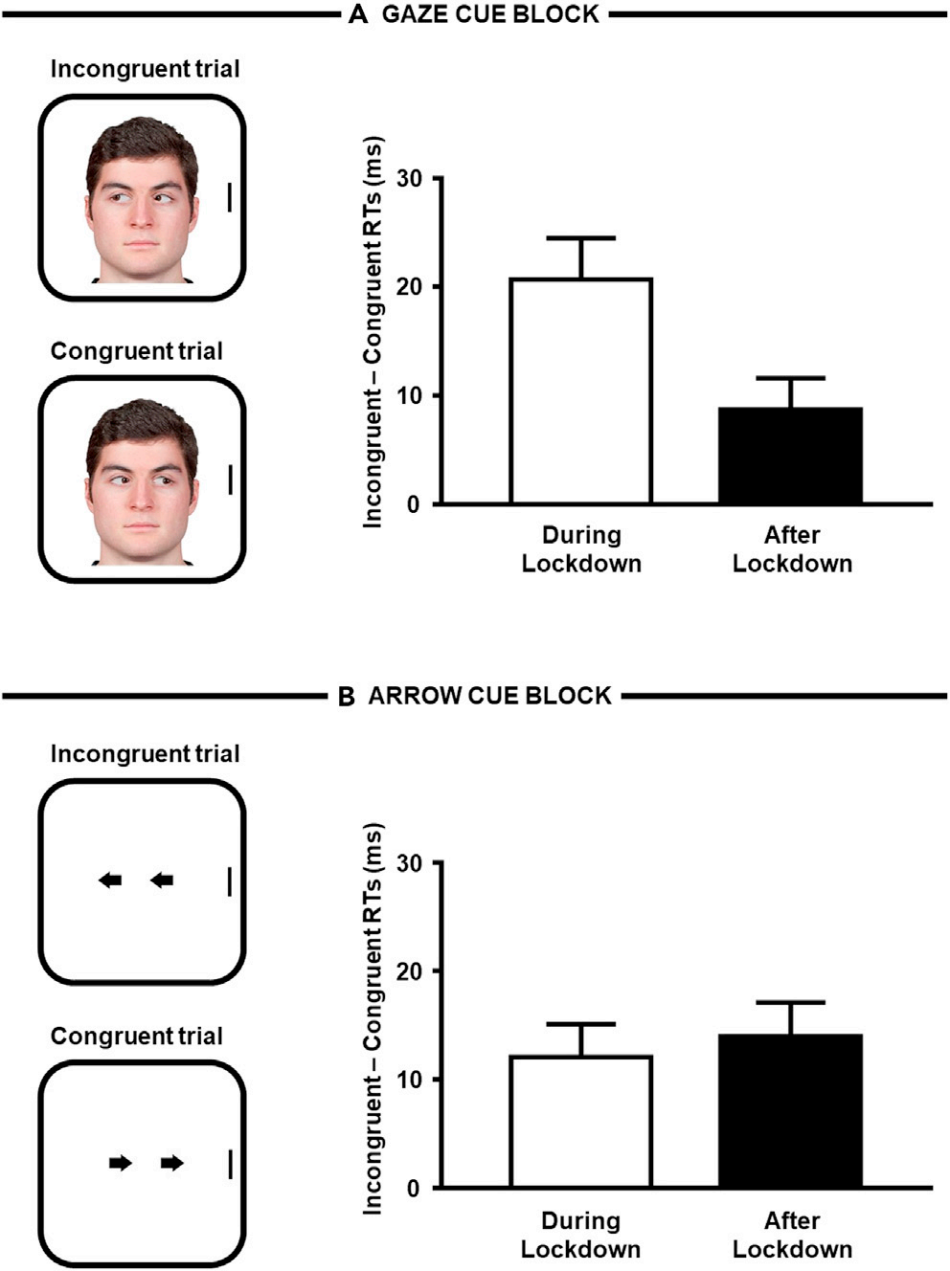
Main results from the spatial-cueing task of the main experiment (A) Magnitude of the gaze-cueing effect (i.e., incongruent-congruent RTs) was greater during the lockdown than after the lockdown. Error bars are SEM. (B) Magnitude of the arrow-cueing effect (i.e., incongruent-congruent RTs) was similar both during and after the lockdown. Error bars are SEM.

We anticipate here the main results of the experiment, which are also summarized in Figure 2 (see also Table 1). As concerns gaze cueing (i.e., attention driven by gaze cues), reaction times (RTs) for congruent trials were smaller as compared with RTs for incongruent trials both during the lockdown and after the lockdown, but crucially the difference (i.e., the gaze-cueing effect) was greater in the former case. In contrast, as concerns nonsocial orienting of attention (i.e., attention driven by arrow cues), the spatial-cueing effect did not differ during and after the lockdown. A further control experiment ruled out alternative accounts according to which the decreased gaze cueing after the lockdown may simply reflect either a practice-like or a habituation-driven effect due to repeated measurements. More specifically, a gaze-cueing paradigm was administered online to a novel sample of participants (N = 30) in a first temporal phase (from June 9^th^ to June 16^th^ 2021). They were invited to complete a second phase after about two weeks (from June 30^th^ to July 11^th^ 2021). Critically, no lockdown was active in either phase. Twenty-five participants took part in both phases. If practice (or habituation) was the driving factor in the main experiment, then one should expect to observe a similar reduction of the gaze-cueing effect also in this control experiment when comparing the magnitude of the phenomenon from the first with the second temporal phase. Crucially, unlike the main experiment, the gaze-cueing effect was found to have the same magnitude irrespective of temporal phase.

**Table 1:** Spatial-cueing task data of the main experiment

		During lockdown		After lockdown	
		Congruent	Incongruent	Congruent	Incongruent
**Gaze cues**	**RTs (ms)**	572 (10.22)	592 (11.47)	547 (10.37)	556 (9.73)
	**% Errors**	5.10 (0.58)	5.47 (0.5)	4.76 (0.51)	5.82 (0.57)
**Arrow cues**	**RTs (ms)**	575 (9.21)	587 (9.99)	557 (10.33)	571 (10.18)
	**% Errors**	4.66 (0.45)	5.50 (0.58)	5.26 (0.57)	5.35 (0.59)

Mean RTs and percentage of errors observed in all the experimental conditions for the participants who took part in both phases. Values in parentheses are SEM.

At the end of the spatial-cueing task of the main experiment, participants were also administered a questionnaire that included two sections. The first section (amount of social interactions; 4 items) was aimed to assess whether the social life of respondents was actually different during and after the lockdown. As expected, during the lockdown, participants were less likely to leave their houses, whereas they engaged in more intensive online interactions. The second section of the questionnaire (COVID-19 distress; 4 items) was aimed to assess how stressful the restrictions and social isolation due to COVID-19 were perceived to be. Critically, the subjective perception of distress during the lockdown period was significantly correlated with the strength of gaze cueing: the more the participants were uneasy for the impact that the lockdown had on their social life, the more they followed the gaze of other individuals. The subjective perception of distress was relatively high both during and after the lockdown.

## Results

### Main experiment

#### Analyses of the spatial-cueing task

Missing responses (0.58% of trials) were very rare, whereas trials in which a wrong response was provided (5.24% of trials) were analyzed separately. Correctly-responded trials with a latency smaller than 150 ms or greater than 1500 ms (0.17% of trials) were considered outliers and therefore discarded. Mean RTs and mean percentage of errors are reported in Table 1.

A repeated-measures ANOVA, with congruency (2: congruent versus incongruent), cue (2: gaze versus arrow) and temporal phase (2: during lockdown versus after lockdown) as within-participants factors, was used to analyze RTs. The main effect of congruency was significant, *F*(1, 66) = 74.376, p < .001, *η*^*2*^_*p*_ = .530, with smaller RTs on congruent trials (*M* = 563 ms, *SE* = 8.98) than on incongruent trials (*M* = 577 ms, *SE* = 9.24), as well as temporal phase, *F*(1, 66) = 12.611, p < .001, *η*^*2*^_*p*_ = .160, with smaller RTs after the lockdown (*M* = 558 ms, *SE* = 9.77) than during the lockdown (*M* = 582 ms, *SE* = 9.56). Importantly, the three-way congruency × cue × temporal phase interaction was also significant, *F*(1, 66) = 4.308, p = .042, *η*^*2*^_*p*_ = .061. No other significant results emerged (*F*s < 2.662, ps > .108). The three-way interaction was explored through two further ANOVAs conducted as a function of cue type. As for gaze cues, both the main effect of congruency, *F*(1, 66) = 37.464, p < .001, *η*^*2*^_*p*_ = .362, and temporal phase, *F*(1, 66) = 13.911, p < .001, *η*^*2*^_*p*_ = .174, were significant. More importantly, the congruency × temporal phase interaction was also significant, *F*(1, 66) = 6.280, p = .015, *η*^*2*^_*p*_ = .087. Two-tailed paired t tests showed that congruent trials differed from incongruent trials both during the lockdown, *t*(66) = 5.423, p < .001, *d* = .663, *95% CI* [.396, .925], and after the lockdown, *t*(66) = 3.015, p = .004, *d* = .368, *95% CI* [.119, .615], but the difference (i.e., the gaze-cueing effect) was greater in the former case (i.e., during the lockdown; 21 ms versus 9 ms). As for arrow cues, both the main effect of congruency, *F*(1, 66) = 33.987, p < .001, *η*^*2*^_*p*_ = .340, and temporal phase, *F*(1, 66) = 5.243, p = .025, *η*^*2*^_*p*_ = .074, were significant. More importantly, the congruency × temporal phase interaction was nonsignificant, *F*(1, 66) = .197, p = .659, *η*^*2*^_*p*_ = .003, indicating that the arrow-cueing effect did not differ during and after lockdown.

In order to provide evidence that the reduction in gaze-cueing magnitude across phases in the main experiment was not caused by practice, we run additional analyses specifically controlling for average response time. In particular, an ANCOVA with temporal phase (2: during the lockdown versus after the lockdown) as factor was conducted on the magnitude of the gaze-cueing effect (i.e., incongruent-congruent RTs) including the difference between the average RTs across phases as covariate. This latter variable can reasonably capture the overall variation in RTs between the two phases. Briefly, the main effect of temporal phase was still significant (*F* = 5.869, p = .018), confirming that gaze cueing was larger during the lockdown than after the lockdown. Moreover, importantly, the interaction with the covariate was not significant (*F* = 0.147, p = .703). This indicates that our main pattern of findings was not caused by the overall reduction in RTs across the two temporal phases.

The same ANOVA as that used for RTs was used for the analyses of the mean percentage of errors. The main effect of congruency was significant, *F*(1, 66) = 4.042, p = .048, *η*^*2*^_*p*_ = .058, with fewer errors on congruent trials (*M* = 4.94%, *SE* = .39) than on incongruent trials (*M* = 5.54%, *SE* = .45). No other significant results emerged (*F*s < 1.384, ps > .244).

#### Analyses of the questionnaire

Mean values for each item and temporal phase are reported in Table 2. As for the amount of social interactions, two-tailed paired t test analyses revealed that during lockdown participants left their houses less often (item A1; *t*(66) = 8.853, p < .001, *d* = 1.082, *95% CI* [.777, 1.381]), they made more video calls (item C1; *t*(66) = 6.387, p < .001, *d* = .780, *95% CI* [.504, 1.052]), and interacted during video calls with a larger number of people (item D1; *t*(66) = 4.490, p < .001, *d* = .549, *95% CI* [.290, .804]), as compared with the period after the lockdown, whereas the number of flat/house mates remained unchanged (item B1; *t*(66) = .199, p = .843, *d* = .024, *95% CI* [−.215, .264]). No significant correlations were found among the magnitude of either gaze or arrow cueing and the different items assessing actual social behavior (−.124 < *r*s < .066, ps > .209). As for the assessment of the COVID-19 distress, two-tailed paired t tests revealed no significant differences during and after lockdown (*t*s < 1.073, ps > .287). In order to address possible correlations between self-reported COVID-19 distress and attentional response, a single score was computed by averaging responses to the four relevant items during the lockdown, given that they were strongly intercorrelated (Cronbach's *α* = .85, *95% CI* [.789, .890]). Critically, the subjective perception of distress during the lockdown period was significantly correlated with the strength of gaze cueing, *r*(104) = .211, p = .032, *95% CI* [.019, .388]: the more the participants were uneasy for the impact that the lockdown had on their social life, the more they followed the gaze of other individuals. In contrast, no such correlation emerged for arrow cueing, *r*(104) = .018, p = .860, *95% CI* [−.176, .209], thus further corroborating the unique involvement of social factors.

**Table 2:** Questionnaire data

	Amount of social interactions				COVID-19 distress			
	A1	B1	C1	D1	A2	B2	C2	D2
**During lockdown**	3.63 (.30)	3.21 (.14)	5.81 (.20)	2.82 (.16)	4.96 (.26)	5.02 (.27)	6.31 (.26)	4.64 (.28)
**After lockdown**	6.75 (.22)	3.19 (.14)	3.73 (.30)	2.02 (.12)	4.69 (.26)	4.85 (.29)	6.46 (.27)	4.46 (.31)

Mean values for each item of the questionnaire collected during and after lockdown based on the responses of the participants who took part in both phases. Values in parentheses are SEM.

In order to provide further evidence about the robustness of the observed correlation pattern, we conducted additional analyses focused on the subsample of participants who took part in both phases and presumably were therefore more motivated. The pattern was robust and also emerged when considering the subsample of the presumably more motivated participants who took part in both phases of the study. Indeed, the correlation between the subjective perception of distress during the lockdown period and the strength of gaze cueing was significant, *r*(67) = .271, p = .027, 95% CI [.033, .480], whereas the correlation with arrow cueing was not, *r*(67) = −.062, p = .620, 95% CI [−.298, .181].

### Control experiment

#### Analyses of the spatial-cueing task

Data were handled as in the main experiment. In particular, missing responses (0.29% of trials) were very rare, whereas trials in which a wrong response was provided (5.42% of trials) were analyzed separately. Correctly responded trials with a latency smaller than 150 ms or greater than 1,500 ms (0.17% of trials) were considered outliers and therefore discarded. Mean RTs and mean percentage of errors are reported in Table 3.

**Table 3:** Spatial-cueing task data of the control experiment

		First phase		Second phase	
		Congruent	Incongruent	Congruent	Incongruent
**Gaze cues**	**RTs (ms)**	595 (15.47)	614 (17.01)	557 (12.77)	576 (12.84)
	**% Errors**	5.58 (2.10)	8.00 (1.87)	3.5 (0.76)	4.58 (0.8)

Mean RTs and percentage of errors observed in all the experimental conditions for the participants who took part in both phases. Values in parentheses are SEM.

A repeated-measures ANOVA, with congruency (2: congruent versus incongruent) and temporal phase (2: first versus second) as within-participants factors, was used to analyze RTs. The main effect of congruency was significant, *F*(1, 24) = 30.646, p < .001, *η*^*2*^_*p*_ = .561, with smaller RTs on congruent trials (*M* = 576 ms, *SE* = 13.56) than on incongruent trials (*M* = 595 ms, *SE* = 14.38). Temporal phase also yielded a significant main effect, *F*(1, 24) = 22.213, p < .001, *η*^*2*^_*p*_ = .481, with smaller RTs in the second phase (*M* = 567 ms, *SE* = 12.58) than in the first phase (*M* = 605 ms, *SE* = 16.09). Importantly, the two-way congruency × temporal phase interaction was not significant, *F*(1, 24) < .001, p = .980, *η*^*2*^_*p*_ < .001. For completeness, two-tailed paired t tests showed that congruent trials differed from incongruent trials both in the first phase, *t*(24) = 4.181, p < .001, *d* = .836, *95% CI* [.373, 1.287], and in the second phase, *t*(24) = 3.993, p < .001, *d* = .799, *95% CI* [.341, 1.244], and their difference (i.e., the gaze-cueing effect) was identical in both phases (i.e., 19 ms; see also Table 3).

The same ANOVA as that used for RTs was used for the analyses of the mean percentage of errors. The main effect of congruency was significant, *F*(1, 24) = 7.934, p = .010, *η*^*2*^_*p*_ = .248, with fewer errors on congruent trials (*M* = 4.54%, *SE* = 1.07) than on incongruent trials (*M* = 6.29%, *SE* = 1.09). No other significant results emerged (*F*s < 1.865, ps > .185). The data of the control experiment further show that practice has an overall impact on absolute RTs but it does not influence the magnitude of gaze cueing.

## Discussion

The dramatic experience of the COVID-19 lockdown provided nonetheless a unique opportunity to investigate the potential impact of social isolation on basic cognitive processes in a highly ecological setting. In so far, the impact of social disconnection has been basically investigated with case studies (Choukér and Stahn, 2020), with correlational studies involving lonely individuals (Cacioppo and Hawkley, 2009), or by triggering specific instances of social rejection (Wilkowski et al., 2009). However, the COVID-19 lockdown is likely to have determined a peculiar constellation of destabilizing feelings characterized by the sudden interruption of social contact and temporal uncertainty about the possibility to re-engage in a face-to-face relational modality.

Here, we addressed social attention, a basic cognitive ability that is considered as one of the micro-level components of social interaction (Colombatto et al., 2020). Our results showed that the magnitude of the gaze-cueing effect was greater during the lockdown than after the lockdown, whereas no such difference emerged for the magnitude of the arrow-cueing effect. Because one may argue that the reduction in gaze cueing simply reflected a practice-like effect (i.e., reduced gaze-cueing magnitude after repeated testing), we carried out a control experiment with a new sample of participants who took part in a gaze-cueing paradigm in two distinct temporal phases during which no lockdown was applied. It is important to note that the temporal interval in between the two temporal phases was much shorter (2 weeks apart) in the control experiment with respect to the main experiment (almost 6 months apart). This, if any, should have increased the likelihood to detect practice-like effects. If practice was the key factor shaping the results of the main experiment, then a similar reduction of the gaze-cueing effect could be expected also in the control experiment. Although any conclusion should be taken with caution due to the differences between the two experiments, because the results showed that gaze-cueing magnitude in the control experiment was unaffected by repeated measurement, then the practice-like account can be reasonably ruled out.

One may also wonder whether the presumable decrease of eye contact exchange associated with virtual meetings (as opposed to meetings in person) may have affected gaze cueing. Our data showed no correlation between items (i.e., items C1 and D1) assessing video call frequency (i.e., virtual interaction contexts, also see Risko et al., 2016) in the lockdown phase and the magnitude of gaze cueing during the lockdown. This, in turn, suggests that a more frequent participation in virtual meetings was not associated with variations in the magnitude of gaze cueing in the present data.

Importantly, the magnitude of the gaze-cueing effect during the lockdown was positively correlated with subjective measures of distress due to social isolation. The more the respondents reacted in a negative way to the lack of social interactions, the more they were sensitive to the gaze direction of other individuals. This finding was further corroborated by an exploratory correlation analysis between the difference score in item A1 (frequency with which participants left their houses) during and after the lockdown and the difference in gaze cueing during and after the lockdown. Interestingly, a significant inverse correlation was found between the two indexes (*r*(67) = −.253, p = .039, *95% CI* [−.465, −.014]). This suggests that the more the participants were isolated during the lockdown as compared with after the lockdown, the stronger the difference in gaze cueing across the two phases was (i.e., higher gaze cueing during the lockdown as compared with after the lockdown). In sharp contrast, no correlation emerged for arrow cueing (*r*(67) = .202, p = .102, *95% CI* [−.041, .421]) and, if anything, it had an opposite direction. Taken together, these data suggest that lockdown affected social attention, inducing a greater responsiveness to eye-gaze stimuli of other individuals.

Social isolation has been shown to be related to a host of psychological aspects, including irritability, insomnia, and stress (Brooks et al., 2020). Experimental studies conducted with animal models have demonstrated that social isolation results in an increased affiliation behavior (Ikemoto and Panksepp, 1992; Latané and Steele, 1975). Recent evidence has suggested that social isolation can be reflected in a craving-like brain response to social stimuli akin to that displayed toward food when hungry (Inagaki et al., 2016; Tomova et al., 2020). Coupled with the results showing that isolated rats prefer social interaction rewards rather than food reward (Ikemoto and Panksepp, 1992), this strongly points to the conclusion that social affiliation can be considered as an “innate need.” This is also supported by the analysis of the brain structures of lonely individuals who show differences in the so-called “default network,” likely reflecting chronic more intense activities related to mentalizing, reminiscing, and imagination (Spreng et al., 2020) aimed at filling the social void they are experiencing. The default network is also known to be involved in the representation of other individuals and in the processing of their intentions (Mars et al., 2012). Forced conditions of social disconnection, such as those due to the Covid-19 lockdown, may have thus prompted to be particularly attuned to the nonverbal behaviors displayed by other individuals and affected the more basic processes subtending the ability to infer mental states and intentions of others, such as social attention probed by the gaze-cueing effect (Capozzi and Ristic, 2018). Overall, current findings are consistent with the presence of a homeostatic system that responds to the aversive signal represented by the lack of social interactions by promoting appropriate responses at both the behavioral and cognitive level aimed at supporting the re-establishment of positive social contact (Gardner et al., 2005; Matthews and Tye, 2019).

To conclude, this work showed that gaze cueing of attention was increased during COVID-19 lockdown. Despite the lockdown has proved a successful measure to lower spreading of COVID-19 (Della Rossa et al., 2020; Fazio et al., 2021), it also led to unwanted secondary effects at the psychological level. Our results indicate that the social life restrictions associated with this confinement can also impact important aspects of human cognition and attention. This suggests that a stronger effort should be put in the investigation of the strategies most suited to represent a viable compromise between the positive outcomes of social distancing and the negative consequences due to social disconnection (Block et al., 2020). This is particularly relevant, given that the world is currently experiencing a new wave of the pandemic and the threat of genetic mutations of the virus, and many nations might enforce novel lockdown restrictions.

### Limitations of the study

The present study offers a unique attempt to investigate the effects of social isolation in the context of a highly ecological and naturalistic context. This, however, came with a cost, in that the study relied on a quasi-experimental design. Hence, no controlled manipulation was employed. As a result, the present findings should be interpreted with caution, given that uncontrolled factors may have played a role in shaping the results. The control experiment addressed one major possible alternative explanation, but other variables might have contributed to affect the responses in the present study. Sample size also represents a potential limitation of the study, because it was determined based on what was logistically feasible during the lockdown, and it had to be stopped with the end of the lockdown. Our goal was to test as many participants as possible, the only constraint being that data collection had to finish before strict lockdown restrictions were softened. Another related limitation is concerned with the drop-out of participants between the first and the second phase of the study.

## STAR★Methods

### Key resources table

**Table:** 

REAGENT or RESOURCE	SOURCE	IDENTIFIER
**Deposited data**		

Raw data and experiment code	This study	https://doi.org/10.17605/OSF.IO/ACNB9

**Software and algorithms**		

PsychoPy 2020.1.2	Open Science Tools Ltd.	https://psychopy.org/index.html
Pavlovia platform 2020.1	Open Science Tools Ltd.	https://pavlovia.org/
JASP 0.14.1	Eric-Jan Wagenmakers, University of Amsterdam	https://jasp-stats.org/

### Resource availability

#### Lead contact

Further information and requests for resources should be directed to and will be fulfilled by the Lead Contact, Mario Dalmaso (mario.dalmaso@unipd.it, mario.dalmaso@gmail.com).

#### Materials availability

This study did not generate any new unique reagents.

### Experimental model and subject details

#### Participants

A convenient sample of undergraduate students at the University of Padova, Italy, took part in the study on a voluntary basis. All of the participants provided an informed consent. The study was approved by the Ethics Committee for Psychological Research at the University of Padova.

As for the main experiment, the initial sample of respondents during the lockdown was composed of 104 individuals (*Mean age* = 24.85 years, *SD* = 7.35, 77 females and 27 males). The sample of respondents who took part in both phases (i.e., both during and after the lockdown) was composed of 67 individuals (*Mean age* = 25.78 years, *SD* = 7.97, 50 females and 17 males).

As for the control experiment, the initial sample of respondents during the first phase was composed of 30 individuals (*Mean age* = 32.03 years, *SD* = 11.39, 26 females and 4 males). The sample of respondents who took part in both phases (i.e., first and second phase) was composed of 25 individuals (*Mean age* = 32.28 years, *SD* = 11.18, 23 females and 2 males).

### Method details

#### Spatial-cueing task

Examples of stimuli and trials are illustrated in Figure 1. As for the social stimuli, three male and three female faces (about 300 px width × 450 px height) were extracted from the MR2 database (Strohminger et al., 2016), which contains high resolution photos of real individuals. Because ethnicity is known to affect gaze cueing (e.g., Zhang et al., 2021a; 2021b), only faces belonging to the same ethnicity of the participants (i.e., White faces) were used. For each face, there were three different versions: one with direct gaze (the original photo), one with gaze averted leftwards and one with gaze averted rightwards (these last two pictures were created through photo editing). These stimuli were effective in eliciting a reliable gaze-cueing effect in a previous study (Strachan et al., 2017). As for the control symbolic stimuli, a figure was created with two black arrows. Also in this case, there were three different versions: one with headless arrows, one with both arrows pointing leftwards and one with both arrows pointing rightwards. Importantly, both eyes and arrows covered the same area (about 55 px width × 40 px height). All stimuli appeared on a white background. The whole experiment was programmed with PsychoPy and delivered online through Pavlovia, which allow to collect both precise and reliable behavioral data (Bridges et al., 2020).

Faces and arrows cueing stimuli were presented in two distinct blocks, randomly selected across participants. Each trial started with a black fixation cross (0.1 normalized units) for 500 ms. Then, depending on the selected block, a face with direct gaze or the two headless arrows appeared, centrally, for 900 ms. Then, the central face looked either leftwards or rightwards or, as for the two arrows, these pointed either leftwards or rightwards. After 200 ms (i.e., Stimulus Onset Asynchrony, SOA), a target appeared either on the left or on the right side of the screen (± 0.8 normalized units from the center of the screen) until a manual response was made, or until timeout (2000 ms), whichever came first. The target consisted of a black line segment (40 px width × 12 px height) appearing either vertically or horizontally. Participants were instructed to look at the center of the screen for the whole duration of the trial and to discriminate, as fast and accurate as possible, the line segment orientation by means of a keypress (“f” or “k” key). The association between keys and line segment orientation was randomly selected across participants. They were also asked to ignore gaze/arrow direction since it was not informative as concerns the location of the upcoming target. In case of a wrong or a missed response, a visual feedback (the words “NO” or “TOO SLOW”, respectively; Arial font, black color, 0.1 normalized units) was provided at the center of the screen for 500 ms. For both faces and arrows, a practice block (10 trials) was followed by an experimental block (96 trials: 48 spatially congruent trials and 48 spatially incongruent trials for each type of cue). Hence, each participant responded to 192 experimental trials in total. Each condition was randomly selected and presented for an equal number of times.

As concerns the control experiment, the experimental paradigm was the same as in the main experiment, but only face stimuli were used (i.e., there were 96 experimental trials in total for each participant).

#### Questionnaire

In the main experiment only, the spatial-cueing task was followed by a questionnaire. Each item appeared centrally (Arial font, black color, 0.05 normalized units) and participants were instructed to read it and to provide a manual response by pressing the number key corresponding to their response. There was no time limit for responding. The questionnaire items are reported below.

Section 1: Amount of social interactions

A1) In this period of coronavirus-related restrictive measures, on average, how often are you used to leave your house?

1. Never
2. Once a month
3. Once every three weeks
4. Once every two weeks
5. Once a week
6. Two-to-three times a week
7. Four-to-six times a week
8. Once a day
9. Several times a day

B1) How many people currently live with you in your house/apartment?

1. I am alone
2. I live with another person
3. I live with two other people
4. I live with three other people
5. I live with more than three other people

C1) In this period of restrictive measures related to the coronavirus, on average, how many times do you video call, through Skype or other software (e.g., Zoom, WhatsApp, etc.), friends, relatives, or other people with whom you have a personal relationship?

1. I do not make use of video calls
2. Once a month
3. Once every three weeks
4. Once every two weeks
5. Once a week
6. Two-to-five times a week
7. Once a day
8. Two-to-three times a day
9. More than four times a day

D1) In this period of restrictive measures related to the coronavirus, on average, how many different people (including your friends, relatives, or other people with whom you have a personal relationship) do you video call every week through Skype or with other software (e.g., Zoom, WhatsApp, etc.)?

1. I do not make use of video calls
2. One-to-three different people per week
3. Four-to-six different people per week
4. Seven-to-ten different people per week
5. Eleven-to-fifteen different people per week
6. Sixteen-to-twenty different people per week
7. More than twenty people per week

Section 2: COVID-19 distress

A2) "How much distress are you experiencing during this period of social isolation due to the coronavirus restrictive measures?"

**Table:** 

Not at all								Very much
1	2	3	4	5	6	7	8	9

B2) "I can no longer bear the restrictive measures related to the coronavirus"

**Table:** 

Not at all								Very much
1	2	3	4	5	6	7	8	9

C2) "My strongest desire now is to get back to my normal life"

**Table:** 

Not at all								Very much
1	2	3	4	5	6	7	8	9

D2) "I feel that the lack of social interactions is draining me"

**Table:** 

Not at all								Very much
1	2	3	4	5	6	7	8	9

### Quantification and statistical analysis

Both descriptive (i.e., mean, SEM) and inferential statistics (i.e., ANOVA, t-test, correlation; alpha level was set at .05) reported in this work were conducted with JASP software (freely available at https://jasp-stats.org/), and can be found in the main text, in the three tables, and in Figure 2.

## Data Availability

The study has not been preregistered. The data generated during this study and the experiment code are publicly available at OSF, https://doi.org/10.17605/OSF.IO/ACNB9. Any additional information required to reanalyze the data reported in this paper is available from the lead contact upon request.
